# Association of Patient Proximity to Dermatologic Care With Melanoma Stage at Diagnosis and Outcome

**DOI:** 10.1001/jamanetworkopen.2022.52698

**Published:** 2023-01-25

**Authors:** Ailynna Chen, Caitlyn S. Grubbs, Faraaz S. Zafar, Bradley T. Loeffler, Sarah L. Mott, Margaret Carrel, Jennifer G. Powers

**Affiliations:** 1 Carver College of Medicine, University of Iowa, Iowa City; 2 College of Liberal Arts and Science, University of Iowa, Iowa City; 3 Holden Comprehensive Cancer Center, University of Iowa Hospitals and Clinics, Iowa City; 4 Department of Dermatology, University of Iowa Hospitals and Clinics, Iowa City

## Abstract

This cohort study evaluates the association of proximity to dermatologic clinicians with stage at diagnosis and cancer-specific survival among adults with cutaneous melanoma in Iowa.

## Introduction

Limited geographic proximity to care has been associated with delayed melanoma diagnosis and higher mortality, and rural populations may be especially susceptible.^1,2,3,4^ However, previous studies did not include advanced practice practitioners (APPs), and their role in access to dermatologic care remains poorly understood. We aimed to determine the association between proximity to dermatologic clinicians and melanoma stage at diagnosis and cancer-specific survival (CSS) in Iowa, a largely rural state.

## Methods

A retrospective cohort study was conducted using data from the Surveillance, Epidemiology, and End Results registry (eMethods in Supplement 1). The University of Iowa Human Subjects Office/Institutional Review Board deemed this study as non–human participant research. We followed the STROBE reporting guideline.

Adults in Iowa with a single lifetime cutaneous melanoma diagnosis between January 2009 and December 2018 were included. Locations of full-time dermatologic clinicians (≥3 days/week) from 2018 to 2020, including physicians and APPs (nurse practitioners and physician assistants), were identified through search engines, telephone calls to clinics, and cross-referencing data registries.^5,6^ For each zip code tabulation area (ZCTA) and clinician type (physicians, APPs, or combined), patient proximity to care was estimated by calculating road travel distance, time from the ZCTA centroid to nearest clinician, and clinician rate per 100 000 population. We categorized travel distance as less than 50 miles or 50 miles or more, time as less than 60 minutes or 60 minutes or more, and clinician rate as 0 or not 0.

Multinomial logistic and Cox proportional hazards regression models were used to evaluate the association of demographic, clinical, and geographic characteristics with stage and CSS. Demographic and clinical characteristics associated with the outcome on univariable analysis and year of diagnosis were included in a multivariable model. Each geographic variable was added to the multivariable model and assessed for significance. For CSS, time was calculated from diagnosis to death from cancer. Patients who died of other causes were censored at death; patients who were still alive were censored at last follow-up. Statistical testing was 2-sided and assessed for significance at the 5% level using SAS 9.4 (SAS Institute).

## Results

We identified 5967 patients (2969 females [49.8%], 2998 males [50.2%]; mean [SD] age, 57.9 [16.9] years). Patient characteristics are described in Table 1. At diagnosis, 4978 patients (83.4%) had localized, 619 (10.4%) had regional, and 251 (4.2%) had distant disease. Of 1249 deaths, 616 (49.3%) were due to cancer. Mean clinician density per 100 000 population was 4.4 (2.1 physicians, 2.3 APPs). Most patients lived in a ZCTA without a clinician (4350 [72.9%]) but were less than 50 miles’ (5591 [93.7%]) or less than 60 minutes’ (5567 [93.3%]) travel from the nearest clinician. Similar results were observed when stratified by clinician type.

**Table 1.** Demographic and Clinical Characteristics of Study Population

Variable	No. (%)
No.	5967
Age, mean (SD), y	57.9 (16.9)
Sex	
Female	2969 (49.8)
Male	2998 (50.2)
Race	
White	5705 (95.6)
Other race^a^	9 (0.2)
Missing data	253 (4.2)
Ethnicity	
Hispanic White	19 (0.3)
Non-Hispanic White	5488 (92.0)
Other ethnicity^a^	42 (0.7)
Missing data	418 (7.0)
Marital status	
Married	3535 (59.2)
Not married	1433 (24.0)
Missing data	999 (16.7)
Health insurance status	
No	117 (2.0)
Yes	4764 (79.8)
Missing data	1086 (18.2)
Melanoma stage	
Localized	4978 (83.4)
Regional	619 (10.4)
Distant	251 (4.2)
Missing data	119 (2.0)
Receipt of surgery	
No	510 (8.5)
Yes	5457 (91.5)
Receipt of radiation	
No	5777 (96.8)
Yes	190 (3.2)
Receipt of chemotherapy	
No	5850 (98.0)
Yes	117 (2.0)
Receipt of biological response modifiers	
No	5689 (95.3)
Yes	278 (4.7)

^a^ Other race and ethnicity included American Indian or Alaska Native, Asian or Pacific Islander, and Black, each of which included fewer than 5 patients.

On multivariable analysis, presence of a full-time clinician in the ZCTA was not associated with melanoma stage at diagnosis (regional vs localized odds ratio [OR], 0.90 [95% CI, 0.74-1.09]; distant vs localized OR, 1.07 [95% CI, 0.80- 1.45]) (Table 2). Presence of a full-time clinician in the ZCTA was not associated with CSS (hazard ratio, 1.07; 95% CI, 0.88-1.29) (Table 2). Both findings were consistent across travel distance, travel time, full-time clinician presence, and clinician type.

**Table 2.** Association Between Covariates and Melanoma Stage at Diagnosis and Cancer-Specific Survival

Variable	Stage at diagnosis, OR (95% CI)^a^		CSS, HR (95% CI)^b^
	Regional vs localized	Distant vs localized	
Presence of a full-time clinician in ZCTA^c^^,^^d^			
No	1 [Reference]	1 [Reference]	1 [Reference]
Yes	0.90 (0.74-1.09)	1.07 (0.80-1.45)	1.07 (0.88-1.29)
Sex			
Male	1 [Reference]	1 [Reference]	1 [Reference]
Female	0.72 (0.60-0.86)	0.49 (0.37-0.65)	0.62 (0.52-0.75)
Married status			
Not married	1 [Reference]	1 [Reference]	1 [Reference]
Married	0.66 (0.55-0.79)	0.64 (0.48-0.84)	0.60 (0.50-0.71)
Age (reference: units = 1 y)	1.00 (1.00-1.01)	1.01 (1.01-1.02)	1.03 (1.03-1.04)
Year of diagnosis (reference: units = 1 y)	1.00 (0.97-1.03)	1.00 (0.96-1.05)	0.96 (0.92-0.99)
Melanoma stage			
Localized	NA	NA	1 [Reference]
Distant	NA	NA	20.81 (15.47-27.98)
Melanoma stage			
Localized	NA	NA	1 [Reference]
Regional	NA	NA	5.64 (4.58-6.96)
Receipt of surgery			
No	NA	NA	1 [Reference]
Yes	NA	NA	0.89 (0.68-1.16)
Receipt of radiation			
No	NA	NA	1 [Reference]
Yes	NA	NA	1.91 (1.51-2.42)
Receipt of chemotherapy			
No	NA	NA	1 [Reference]
Yes	NA	NA	2.70 (2.07-3.53)
Receipt of biological response modifier			
No	NA	NA	1 [Reference]
Yes	NA	NA	0.94 (0.74-1.19)

Abbreviations: CSS, cancer-specific survival; HR, hazard ratio; NA, not applicable; OR, odds ratio; ZCTA, zip code tabulation area.

^a^ Complete case analysis was based on N = 4887.

^b^ Complete case analysis was based on N = 4883.

^c^ Presence of a full-time clinician in the ZCTA was not associated with melanoma stage at diagnosis after adjusting for age, sex, marital status, and year of diagnosis.

^d^ Presence of a full-time clinician in the ZCTA was not significantly associated with CSS after adjusting for age, sex, marital status, diagnosis year, melanoma stage, receipt of surgery, radiation, chemotherapy, and biological response modifiers.

## Discussion

Proximity to dermatologic care was not associated with melanoma stage at diagnosis or survival across multiple geographic measures. Previous studies^1,2,3^ varied in geographic measures used, and proximity’s role in access may be decreasing. Although most ZCTAs had no dermatologic clinicians, more than 93% of patients still lived within driving distance. Additionally, APPs composed more than 50% of the workforce and may be a source of extended access.

A study limitation was using clinician presence from 2018 to 2020 as a proxy because it was unfeasible to accurately capture presence for each diagnostic year. There was little evidence to suggest clinician presence changed significantly to introduce bias. Lack of proximity to clinicians may not be as critical a barrier to care as previously thought, warranting further studies.

## Notes

Supplement 1.
eMethods.

Supplement 2.
Data Sharing Statement
