# Supplementary material for: Association of Patient Proximity to Dermatologic Care With Melanoma Stage at Diagnosis and Outcome
Source: JAMA Netw Open. 2023 Jan 25;6(1):e2252698. doi: 10.1001/jamanetworkopen.2022.52698 (PMC10187484; doi:10.1001/jamanetworkopen.2022.52698)
Supplement: Supplement 1. — eMethods. [file jamanetwopen-e2252698-s001.pdf]

Supplementary Online Content

Chen A, Grubbs CS, Zafar FS, et al. Association of patient proximity to dermatologic care with melanoma stage at diagnosis and outcome. *JAMA Netw Open*. 2023;6(1):e2252698. doi:10.1001/jamanetworkopen.2022.52698

eMethods.

This supplementary material has been provided by the authors to give readers additional information about their work.

© 2023 Chen A et al. *JAMA Network Open.*

eMethods.

Clinical and demographic characteristics obtained from the Surveillance, Epidemiology, and End Results (SEER) program through the Iowa Cancer Registry include melanoma stage at diagnosis (localized/regional/distant disease), sex, age at diagnosis, race, ethnicity, marital status, insurance status, zip code and county of residence, treatments received (receipt of surgery/chemotherapy/radiation/biologic response modifiers), year of diagnosis, vital status and whether death was due to cancer. Race and ethnicity data obtained through the Iowa Cancer Registry was extracted from patient electronic health records. Cancer-specific survival was defined as no death due to cancer from the date of diagnosis to the date of last follow-up. The exact date of last follow-up varied between patients but the overall vital status for the cancer registry was update through the end of 2021. Patients who died due to other causes were censored at death. Patients that were still alive were censored at last follow-up.

© 2023 Chen A et al. *JAMA Network Open.*
