# Supplementary material for: Association of Patient Proximity to Dermatologic Care With Melanoma Stage at Diagnosis and Outcome
Source: JAMA Netw Open. 2023 Jan 25;6(1):e2252698. doi: 10.1001/jamanetworkopen.2022.52698 (PMC10187484; doi:10.1001/jamanetworkopen.2022.52698)
Supplement: Supplement 2. — Data Sharing Statement [file jamanetwopen-e2252698-s002.pdf]

Data Sharing Statement

Chen. Association of Patient Proximity to Dermatologic Care With Melanoma Stage at Diagnosis and Outcome. *JAMA Netw Open*. Published January 25, 2023. doi:10.1001/jamanetworkopen.2022.52698

Data
Data available: No

Additional Information
Explanation for why data not available: Melanoma patient data was acquired through the Iowa Cancer Registry and will not be made publicly available without their mission. Individual dermatology provider data we acquired contain their full name and practice address and will not be made publicly available without their permission. If necessary, we can provide the data upon request on a case-by-case basis.
